# FBXO11 is a candidate tumor suppressor in the leukemic transformation of myelodysplastic syndrome

**DOI:** 10.1038/s41408-020-00362-7

**Published:** 2020-10-06

**Authors:** Michael Schieber, Christian Marinaccio, Lyndsey C. Bolanos, Wendy D. Haffey, Kenneth D. Greis, Daniel T. Starczynowski, John D. Crispino

**Affiliations:** 1 Robert H. Lurie Comprehensive Cancer Center, Division of Hematology/Oncology, Department of Medicine, Feinberg School of Medicine, Northwestern University, Chicago, IL USA; 2 Division of Experimental Hematology and Cancer Biology, Cincinnati Children’s Hospital Medical Center, Cincinnati, OH, USA; 3 UC Proteomics Laboratory, University of Cincinnati, Cincinnati, OH USA; 4 Department of Cancer Biology, University of Cincinnati, Cincinnati, OH USA; 5 Department of Pediatrics, University of Cincinnati, Cincinnati, OH USA; 6 Present Address: Division of Experimental Hematology, Department of Hematology, St. Jude Children’s Hospital, Memphis, TN USA

**Keywords:** Oncogenesis, Myelodysplastic syndrome

## Abstract

Myelodysplastic syndrome (MDS) is a heterogeneous myeloid malignancy characterized by blood cell morphological dysplasia, ineffective clonal hematopoiesis, and risk of transformation to secondary acute myeloid leukemia (sAML). A number of genetic abnormalities have been identified in MDS and sAML, but sensitive sequencing methods can detect these mutations in nearly all healthy individuals by 60 years of age. To discover novel cellular pathways that accelerate MDS and sAML, we performed a CRISPR/Cas9 screen in the human MDS-L cell line. We report here that loss of the F-Box protein FBXO11, a component of the SCF ubiquitin ligase complex, confers cytokine independent growth to MDS-L cells, suggesting a tumor suppressor role for FBXO11 in myeloid malignancies. Putative FBXO11 substrates are enriched for proteins with functions in RNA metabolism and, of note, spliceosome mutations that are commonly found in MDS/sAML are rare in patients with low FBXO11 expression. We also reveal that loss of FBXO11 leads to significant changes in transcriptional pathways influencing leukocyte proliferation, differentiation, and apoptosis. Last, we find that FBXO11 expression is reduced in patients with secondary AML. We conclude that loss of FBXO11 is a mechanism for disease transformation of MDS into AML, and may represent a future therapeutic target.

## Introduction

Myelodysplastic syndrome (MDS) and secondary acute myeloid leukemia (sAML) are bone marrow cancers that arise from clonally derived hematopoietic stem cells (HSCs) that have acquired advantageous DNA alterations through aging or through prior exposure to chemotherapy and/or radiation (therapy-related MDS/AML). Sequencing of large MDS/sAML cohorts has revealed recurrent and overlapping somatic mutations in these diseases. Broadly, these include abnormalities in components of the RNA spliceosome (*SF3B1*, *SRSF2*, and *U2AF1*), proteins involved in epigenetic regulation and histone medication (*TET2*, *IDH1/2*, *EZH2*, and *ASXL1*), transcription factors involved in hematopoietic differentiation (*RUNX1* and *WT1*), and the tumor-suppressor protein *TP53*^1^.

MDS/sAML mutations are also present at significant frequencies in otherwise healthy patients, a phenomenon termed clonal hematopoiesis (CH)^2^. When deep-sequencing techniques are employed, CH can be detected in up to 95% of healthy individuals between 50 and 60 years old^3^. Therefore, acquisition of CH mutations is insufficient to cause disease in most individuals, and other factors must influence how clonal HSCs undergo transformation. There is increasing data supporting a direct relationship between clonal HSC expansion and specific cytokine or regulatory immune cell signatures within the bone marrow environment^4^. For example, *Tet2*-null HSCs exhibit a myeloproliferative phenotype with incomplete penetrance, but display IL-6-dependent proliferation during chronic bacterial infection that can be abrogated by antibiotic therapy^5^. Thus, designing MDS/sAML therapies tailored to the extrinsic factors that create a permissive environment for expansion may be as effective as targeting a somatic mutation acquired in an expanded HSC clone.

In vitro studies of MDS/sAML have been limited due to the paucity of human MDS cell lines, the late spontaneous onset of disease in animal models, and the inability to separate normal and MDS bone marrow elements in primary samples. The human MDS-L cell line, derived from bone marrow mononuclear cells from a 52-year-old male MDS patient, maintains cytokine-dependent growth in vitro, and transplantation of MDS-L cells into irradiated mice leads to an MDS phenotype^6–8^. Moreover, engraftment of MDS-L and onset of disease was accelerated in NOD-scid Il2Rγ^−/−^ (NSGS) mice expressing human cytokines (IL-3, GM-CSF, and SCFA) as compared to NSG, suggesting that cytokine-dependent signals are required for the leukemic cell properties of MDS-L cells in vitro and in vivo. Here, we leveraged the cytokine dependence of MDS-L to perform a genome-wide loss-of-function CRISPR/Cas9 screen to identify critical intrinsic and extrinsic regulators of the leukemic properties of MDS-propagating cells.

One of the top hits identified by the CRISPR/Cas9 screen that conferred cytokine-independent growth of MDS-L cells was the F-box protein, FBXO11. F-box proteins serve as substrate adapters for SKP1–CUL1–F-box complexes that form functional ubiquitin ligases^9^. There are over 60 putative F-box proteins in humans, many still uninvestigated, that have a unique set of substrates and regulate numerous cellular processes. A tumor-suppressor function for FBXO11 was suggested after somatic mutations, and deletions in *FBXO11* were identified in patients with diffuse large B-cell lymphoma (DLBCL)^10^. FBXO11 targets BCL6, a transcription factor critical in B-cell development, for ubiquitin-dependent degradation, and FBXO11 loss leads to upregulation of BCL6, a common finding in DLBCL. Mutations in *FBXO11* have also been identified in 10–20% of Burkitt’s lymphoma and cases of de novo AML, suggesting that it may have a broad role as a tumor suppressor in hematologic malignancies^11,12^. Therefore, we explored a role for loss of FBXO11 in MDS/sAML, and demonstrate that FBXO11 loss imparts a growth advantage to MDS-L cells. We furthermore define the pathways and substrates regulated by FBXO11, and demonstrate decreased expression of FBXO11 in primary patient samples. Together, our data reveal a novel mechanism responsible for the function of MDS-propagating cells, and support therapeutically targeting the FBXO11 pathway in myeloid malignancies.

## Materials and methods

### Cell culture and in vitro cytokine-independent assays

The identity of the MDS-L line was confirmed by STR profiling, and testing for mycoplasma contamination was negative. MDS-L cells were maintained in RPMI medium containing l-glutamine, penicillin/streptomycin, and human recombinant IL-3 at a final concentration of 10 ng/mL (MDS-L media) as previously described^7,13^. To assay for cytokine-independent survival, MDS-L cells were washed three times in PBS to remove residual IL-3 and resuspended in triplicate at a concentration of 125,000 cells/mL in MDS-L media without IL-3. The number of viable cells was quantified on day 6 or 9 using a hemocytometer and trypan blue exclusion.

### CRISPR/Cas9 screening

Cas9-expressing MDS-L cells were established through lentiviral transduction of the lentiCas9-Blast plasmid (Addgene #52962) with 7 days of blasticidin selection at 2 μg/mL. The Brunello Human CRISPR-knockout pooled library (Addgene #73179-LV) was then transduced into Cas9-expressing MDS-L cells with 7 days of puromycin selection at 1 μg/mL^14^. Screening for cytokine-independent survival was performed by passaging 5 × 10^7^ MDS-L cells in IL-3-free MDS-L media for 4 weeks. The surviving cells were then expanded and harvested for genomic DNA. The extracted DNA was barcoded using staggered P5 and index P7 primers (available in Addgene #73196 Sequencing Protocol) and sequenced at the Northwestern University Core Facility to identify enriched sgRNAs (Illumina NextSeq, single-end 75-bp read length, 15–20 M reads per sample). Reads from two independent screens were analyzed using the PinAPL-Py web-based tool and compared to the reads from the parent library for fold enrichement^15^. Raw data from this analysis can be found in the supplementary information.

### FBXO11 cloning methods

On-target FBXO11 editing was confirmed by cloning sgRNAs targeting *FBXO11* into the lentiguide-Puro vector (Addgene #52963), lentiviral transduction of this construct into Cas9-expressing MDS-L cells, and measurement of the reduction in FBXO11 by Western blotting (Novus Biologicals #59826 [FBXO11] Centennial, CO, Santa Cruz #365062 [GAPDH] Dallas, TX). *FBXO11* variants were cloned from human cDNA using the Gibson Assembly Master Mix (New England Biolabs E5510 Ipswich, MA) into the XhoI site of the pRRL.EF1a.MCS.pgk.eGFP-overexpression vector (a gift from Andrew Volk). Point mutations rendering CRISPR resistance to FBXO11-overexpression constructs were introduced using the QuikChange Lightning Site-Directed Mutagenesis Kit (Agilent Technologies #210518 Santa Clara, CA). GFP-expressing MDS-L cells were detectable within 48 h and sorted using FACS. All oligonucleotides used in this paper are provided in the supplemental information (Integrated DNA Technologies, Coralville, IA, Table S1).

### Proteomics screen for FBXO11 ubiquitin substrates

Identification and quantification of ubiquitinated peptides was accomplished by coupling ubiquitin enrichment with a label-free, data-independent acquisition (DIA) mass spectrometry workflow. Specifically, Cas9-expressing MDS-L cells were transduced with a FBXO11 sgRNA and grown in MDS-L media in the presence of IL-3 to ensure growth of control MDS-L cells. Transduced cells were lysed, the proteins were digested with trypsin, and the peptides desalted on a C18 SepPak (Waters Milford, MA). Ubiquitinated peptides were immunoenriched with anti-diGlycine agarose beads using the PTMScan Ubiquitin Proteomics System (Cell Signaling Technology, Danvers, MA) using the vendor protocol. Eluted peptides were analyzed by nano liquid chromatography mass spectrometry (nanoLC–MS)/MS on a Sciex 5600+ TripleTOF mass spectrometer coupled to an Eksigent nanoLC ultra nanoflow high performance liquid chromatography system as reported previously^16^, but with the following instrument parameters. DIA was set with a collection range from 350 to 750 m/z using an 8-m/z isolation window and 1-m/z overlap. Each scan cycle included a 250-ms time-of-flight mass spectrometry spectrum followed by 57 × 50-ms overlapping 8-m/z region for a total duty cycle of 3148 ms. In total, 1681 cycles were collected over the course of the 100-min gradient collection run. Comparative quantification of peptides was achieved using R programing that included VCN normalization, unbiased cluster analysis, principal component analysis, Pearson correlations, and relative ubiquitin peptide changes as described previously^16^.

### RNA sequencing

RNA from three independent experimental replicates was isolated from Cas9-expressing MDS-L cells, FBXO11-knockout MDS-L cells, and FBXO11-knockout MDS-L cells transduced with FBXO11.v4^sm1^ or FBXO11.v1^sm1^ to reconstitute FBXO11 expression (Zymo Research #R2050, Irvine, CA). Cells were grown in the presence of IL-3 to ensure growth of cells under control and FBXO11-reconstitution conditions. Paired-end 150-bp RNA sequencing was performed on an Illumina HiSeq at a depth of 20–30 million reads per sample (Genewiz Inc., South Plainfield, NJ). Sequencing read quality was assessed by FastQC, and reads were aligned to the UCSC human reference genome hg38 using STAR with standard parameters. Read summarization was performed using featureCounts from the Subread package. Differential expression analysis was performed using the EdgeR Quasi-likelihood Tests pipeline with an absolute fold-change and *p* value cutoffs of 1 and 0.05, and pathway analysis was performed on the Metascape platform^17^. Gene expression data are available at Gene Expression Omnibus (accession number GSE156708).

To generate a list of differentially expressed transcripts in SRSF2^mt^ AML, all cases from the BEAT-AML dataset annotated either as splicing-factor wildtype (SF^WT^) or SRSF2^mt^ and data were retrieved using the TCGAbiolinks package and then analyzed as above^18,19^.

### Primary patient sample collection and FBXO11 expression analysis

Samples were collected after informed consent to a prospective tissue and clinical registry that was approved by the Northwestern University Institutional Review Board. All samples, which included peripheral blood and/or bone marrow aspirate, were collected prior to initiation of leukemia-directed therapy, with the exception of hydroxyurea for cytoreduction. The definition of sAML was predefined and included AML with myelodysplastic-related changes or characteristic cytogenetics (AML–MRC), therapy-related AML (tAML), and AML with known antecedent MDS phase. Mononuclear cells were isolated within 24 h via Ficoll density gradient centrifugation (GE Healthcare 17-1440-02) followed by red blood cell lysis (ThermoFisher #00-4300-54, Waltham, MA). Leukemic cells were purified based on either CD34 or CD117 expression using magnetic bead separation (Miltenyi Biotec #130-046-702 and #130-091-332, Auburn, CA) and directly lysed in radioimmunoprecipitation assay (RIPA) buffer to determine FBXO11 expression via Western blotting. Control CD34^+^ cells were collected via plasmapheresis from a healthy donor and directly lysed in RIPA buffer. Densitometry was performed using Image J Software.

### EZH2 splicing analysis

Primary sAML samples were collected from peripheral blood or bone marrow aspiration, and purified via CD34 or CD117 magnetic bead separation, as described above. Cells were immediately lysed in TriZol Reagent (ThermoFisher #15596026) and stored at −80 °C. 500 ng of purified RNA was used for cDNA synthesis (Zymo Research #R2050, ThermoFisher #4368814). 2 μL of the cDNA reaction at 1:5 dilution was added to SYBR green real-time polymerase chain reaction master mix reaction in triplicate for each patient sample (Bio-Rad #1725270, Hercules, CA). The fraction of EZH2 exon inclusion was calculated by dividing the average *C*_t_ value of the EZH2^inc^ transcript by the EZH2^exc^ transcript^20^.

## Results

### CRISPR/Cas9 screen for cytokine-independent growth in MDS-L

**Fig. 1:**
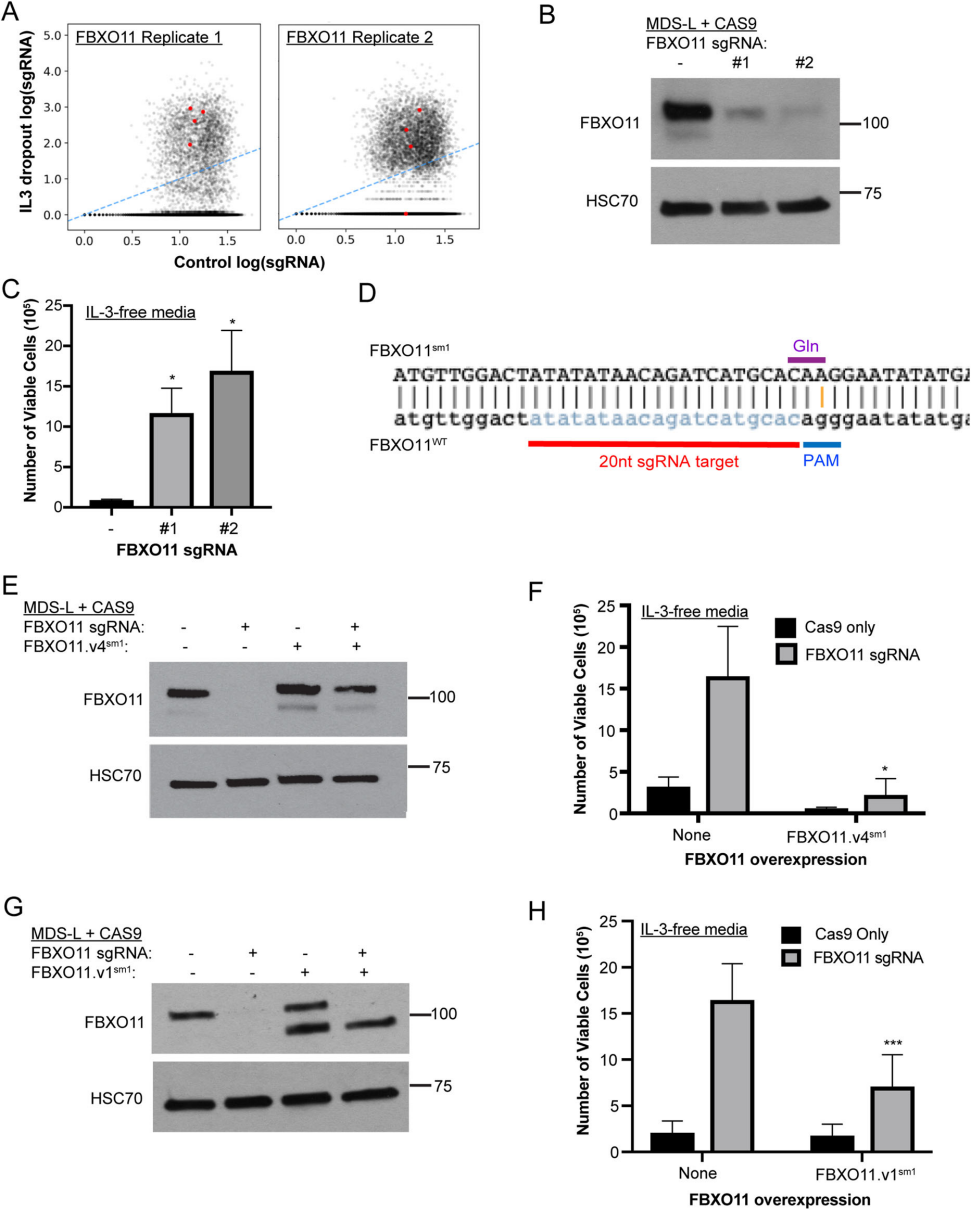
Loss of *FBXO11* promotes cytokine-independent growth of MDS-L cells. **a** Fold enrichment of sgRNAs in cells cultured in IL-3-dropout conditions versus control cells. sgRNAs targeting FBXO11 that were enriched in cells that survived IL-3 withdrawal are highlighted in red. Two biological replicates were performed and are displayed. **b** Western blot analysis of FBXO11 expression in MDS-L/Cas9 cells harboring two independent FBXO11-targeting sgRNAs. HSC70 is shown as a loading control. **c** Numbers of viable MDS-L cells in IL-3-deficient media with or without FBXO11 sgRNA after 6 days of culture. An unpaired two-tailed *t* test was performed to assess for significance between each FBXO11 sgRNA and control conditions (*p < 0.05, error bars represent the SEM of 3 biological replicates). **d** Design of a CRISPR-resistant variant (FBXO11.v4^sm1^). Gray sequence is the sgRNA target sequence. The orange line highlights the introduced synonymous mutation in the CRISPR PAM sequence. The purple line denotes the translated reading frame, which in both variants encodes a Gln (CAG\CAA). **e** Western blot analysis of FBXO11 levels in MDS-L cells with overexpression of the FBXO11.v4^sm1^ CRISPR-resistant allele. HSC70 is shown as a loading control. **f** Numbers of MDS-L cells in IL-3-deficient media with or without FBXO11 sgRNA and exogenous FBXO11v4^sm1^ after 9 days of culture. Note that expression of the FBXO11.v4^sm1^ CRISPR-resistant allele sensitized MDS-L cells to cytokine starvation. An unpaired two-tailed *t* test was performed to assess for significance between FBXO11 sgRNA and FBXO11 sgRNA with FBXO11.v4^sm1^ conditions (*p < 0.05, error bars represent the SEM of 3 biological replicates). **g** Western blot analysis of FBXO11 levels in MDS-L cells with overexpression of the FBXO11.v1^sm1^ CRISPR-resistant allele confirming CRISPR-resistant overexpression of the shorter FBXO11.v1^sm1^. **h** Numbers of MDS-L cells in IL-3-deficient media with or without FBXO11 sgRNA and exogenous FBXO11.v1^sm1^ after 9 days of culture. Note that expression of FBXO11.v1^sm1^ resensitized MDS-L cells to cytokine starvation. An unpaired two-tailed *t* test was performed to assess for significance between FBXO11 sgRNA and FBXO11 sgRNA with FBXO11.v1^sm1^ conditions (***p < 0.001, error bars represent the SEM of 7 biological replicates). An *F* test was used to compare variances between compared groups in Fig. 1d, f, and h and was not significant.

To identify genes and pathways that regulate cytokine-independent growth of MDS-L cells, we performed a whole-genome loss-of-function screen using CRISPR/Cas9. To demonstrate the feasibility of Cas9 editing in MDS-L, we first confirmed expression of FLAG-tagged Cas9 following lentiviral transduction of the lentiCas9-blast plasmid (Supplemental Fig. 1a). Next, to identify genes whose loss promotes cytokine-independent growth of MDS-L cells, we transduced the MDS-L cells expressing Cas9 with the Brunello Human shRNA-knockout library and subjected the resultant puromycin-resistant cells to 4 weeks of IL-3 starvation. The Brunello Human shRNA-knockout library contains 4-guide coverage of each individual gene in addition to nontargeting control sgRNAs for a total of 76 441 pooled guides. After DNA sequencing, the abundance of sgRNAs enriched from two independent biological replicates was compared to the parent library. This analysis revealed 22 candidate genes with an average of 20-fold enrichment in at least two guides. Among these, there was enrichment of three guides for only three genes: FBXO11 (fold change 39.07), PRX (fold change 35.63), and PTEN (fold change 31.46) (Table 1, Fig. 1a, Supplemental Table S2).

**Table 1:** Enriched sgRNAs for genome-wide screen for cytokine-independent growth of MDS-L.

Gene name	Number of significant independent sgRNAs	Average IL-3-dropout counts	Average control counts	Average fold change
BLVRB	2	2728.59	10.52	259.32
C1orf106	2	516.88	16.20	31.90
CCDC97	2	1019.06	8.37	121.68
CORO6	2	590.80	16.56	35.68
CYP2B6	2	849.26	10.83	78.40
CYP2F1	2	5809.56	25.89	224.41
CYP2S1	2	5809.56	8.68	668.93
DLGAP1	2	338.23	6.75	50.09
DMKN	2	817.59	15.53	52.64
FBXO11	3	528.21	13.52	39.07
HACE1	2	514.36	12.36	41.62
JAKMIP2	2	507.59	15.68	32.38
MAP3K10	2	1178.02	17.13	68.76
PEBP4	2	235.65	8.90	26.48
PLD3	2	1618.89	18.63	86.88
PRX	3	603.53	16.94	35.63
PTEN	3	744.11	23.65	31.46
SUPT20H	2	298.75	12.74	23.45
TMEM56	2	375.37	11.69	32.11
WT1	2	512.88	13.17	38.94
ZNF30	2	646.94	10.45	61.91
ZNF555	2	946.00	13.27	71.31

In total, 22 candidate genes were identified with over 20-fold enrichment of two or more sgRNAs. Data are averaged from duplicate cytokine-independent screens.

### FBXO11 is required for cytokine-independent survival of MDS-L cells

We prioritized FBXO11 for further validation as it has been previously described as a tumor suppressor in DLBCL, but its role in MDS or AML has not been investigated. To confirm on-target editing of FBXO11, we synthesized two independent sgRNAs targeting the gene and introduced them into the MDS-L cells with Cas9 and detected significantly reduced FBXO11 expression by Western blotting after 5 days (Fig. 1b). Control MDS-L cells (Cas9 expression only) did not expand in the absence of IL-3 after 6 days; however, deletion of FBXO11 imparted a cytokine-independent expansion of MDS-L cells (Fig. 1c). These findings confirmed our CRISPR/Cas9 screen that loss of FBXO11 is important for the response of MDS-L cells to cytokine-dependent growth.

We next asked whether reconstitution of FBXO11 expression was sufficient to resensitize FBXO11-knockout MDS-L cells to cytokine starvation. Of note, there are two predicted isoforms of FBXO11 based on the presence of an alternative start codon in the mRNA sequence: FBXO11 variant 4 (FBXO11.v4), which encodes a 104-kDa protein and a smaller 94-kDa variant 1 isoform (FBXO11.v1) that lacks the 84-amino acid N-terminal extension (Supplemental Fig. 1B). To rescue FBXO11 expression, we synthesized a CRISPR-resistant FBXO11.v4 cDNA (FBXO11.v4^sm1^) by creating a synonymous mutation in the PAM-recognition sequence of the sgRNA while maintaining a codon encoding glutamine (Fig. 1d). Stable expression of FBXO11.v4^sm1^ was confirmed in MDS-L cells, even in the presence of Cas9 (Fig. 1e). In line with our prediction, FBXO11.v4^sm1^ expression was sufficient to resensitize MDS-L cells to cytokine withdrawal (Fig. 1f). We also tested whether re-expression of the shorter FBXO11.v1 isoform also altered the degree of cytokine dependence. To this end, we introduced an identical PAM-site silent mutation in the cDNA for FBXO11.v1 (FBXO11.v1^sm1^) and confirmed CRISPR-resistant expression by Western blotting (Fig. 1g). We observed that FBXO11.v1^sm1^ expression partially resensitized the MDS-L cells to cytokine withdrawal (Fig. 1h). Based on the comparative size of the endogenous and overexpressed isoforms (Fig. 1e, g), we conclude that FBXO11.v4 is the predominant isoform expressed in MDS-L and its loss promotes cytokine-independent survival.

### FBXO11 substrates are enriched for RNA metabolic pathways

**Fig. 2:**
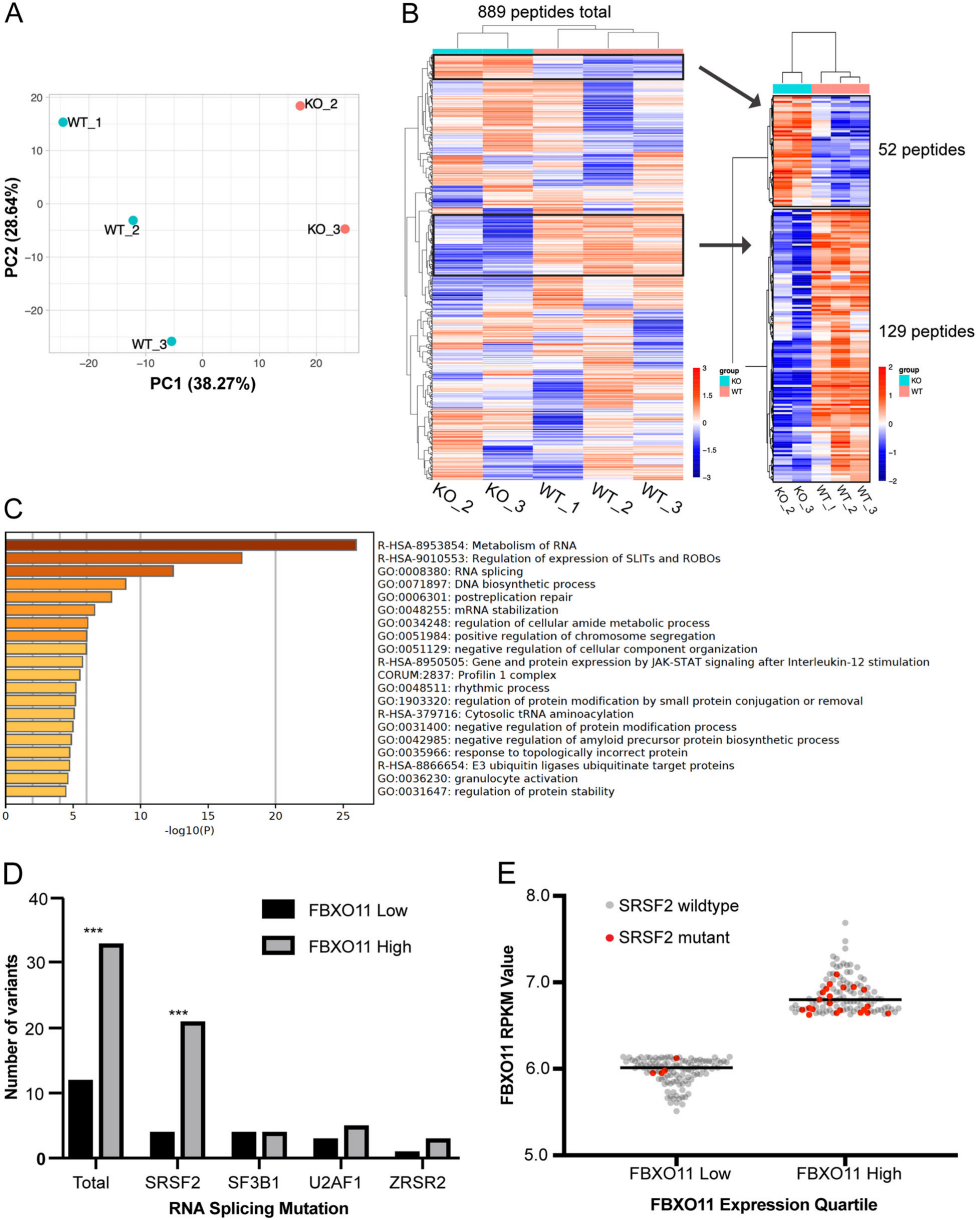
FBXO11 substrates are enriched in RNA metabolic pathways. **a** Principal component analysis of MDS-L wildtype and FBXO11-knockout proteomic samples. **b** Heatmap highlighting 129 peptides showing loss of ubiquitination as would be expected upon loss of FBXO11. **c** Metascape GO pathway analysis of 129 peptides displaying decreased ubiquitination signature in FBXO11-knockout cells. **d** Analysis of spliceosome mutations in the BEAT-AML cohort, stratified by the highest and lowest quartile of FBXO11 expression. A two-sided Fisher’s exact test was employed to assess for significance between FBXO11-low and -high cohorts (***p < 0.001). **e** Plot comparing the number of *SRSF2* variants in cases with the highest and lowest quartile of *FBXO11* mutations.

A tumor-suppressor role for FBXO11 was first discovered in DLBCL, where it is mutated in up to 10% of cases and selectively ubiquitinates the germinal-center transcription factor BCL6 leading to its degradation^10^. However, a role for BCL6 in myeloid leukemia has not been reported. To identify a direct molecular mechanism for the tumor-suppressor-like function of FBXO11 in MDS, we performed a global quantitative ubiquitin-capture proteomic screen in the MDS-L model. Ubiquitinated peptides immunoprecipitated from shFBXO11-MDS-L (KO) or MDS-L cells only expressing Cas9 (WT) were analyzed by mass spectrometry. Principal component analysis revealed clustering of the three WT and two KO biological replicates (Fig. 2a). The dataset detected 2778 unique peptides, which were enriched in 889 ubiquitinated sequences (32% enrichment) in KO cells as compared to WT cells. Of these 889 Ub peptides, 129 displayed a decrease in abundance after FBXO11 knockout, as would be expected for a direct FBXO11 substrate (Fig. 2b, Supplemental Table 3). The 14 ubiquitinated peptides, which displayed a decrease in abundance of 75% or greater, are listed in Table 2. In some cases, multiple peptides for a given protein showed decreased abundance, whereas in others, only one ubiquitinated sequence was identified.

**Table 2:** Candidate genes featuring loss of ubiquitination in *FBXO11*-knockout conditions.

Gene	Ubiquitin peptide sequence	Average WT intensity	Average KO intensity	KO:WT fold change
RHG09	VSGNLAVVQK[UGG]LR	63,726.37	4499.27	0.07
RASF2	LK[UGG]ATDYPLIAR	65,008.48	8262.26	0.13
HNRPC	KSDVEAIFSK[UGG]YGK	137,911.13	21,875.25	0.16
HNRPM	AFITNIPFDVK[UGG]WQSLK	82,112.11	13,131.84	0.16
MATR3	MK[UGG]SQAFIEMETR	67,123.59	11,247.93	0.17
HNRH2	FFSDC[CAM]K[UGG]IQNGTSGIR	56,492.80	11,147.58	0.20
HNRPC	YGK[UGG]IVGC[CAM]SVHK	51,683.47	10,547.67	0.20
PRKDC	WVELAK[UGG]LYR	133,730.22	29,267.87	0.22
PGAM5	HLPGVC[CAM]K[UGG]VSTDLLR	114,705.99	25,494.06	0.22
PUR2	LAQSHHVK[UGG]QVLVAPGNAGTAC[CAM]SEK	32,134.29	7398.63	0.23
RABX5	K[UGG]FFSASSR	31,569.66	7398.21	0.23
ELAV1	LGDK[UGG]ILQVSFK	45,911.79	10,796.57	0.24
PSA5	AIGSASEGAQSSLQEVYHK[UGG]SMTLK	42,106.71	10,356.69	0.25
MYO1G	AK[UGG]VAAMGALQGLR	69,338.58	17,364.96	0.25

In total, 14 candidates displaying greater than 75% reduction in ubiquitination signature upon *FBXO11* knockout. Genes in yellow were detected with the single peptide as shown. The peptide with the most significant decrease in abundance is displayed.

We next performed a Metascape GO pathway analysis of the 129 ubiquitinated peptides with decreased abundance in FBXO11-knockout MDS-L cells and discovered substantial enrichment of proteins involved in RNA processing and metabolism (Fig. 2c). This is relevant because mutations in components of the RNA splicing machinery are common in de novo AML and enriched in patients with MDS^21^. If FBXO11 loss leads to MDS evolution through a common RNA metabolic pathway, we hypothesized that there might be an epistatic relationship between low FBXO11 expression and RNA spliceosome mutations. We analyzed the publicly available BEAT-AML database, which contains predominately cases of de novo AML, and stratified patients to the top and bottom quartile of FBXO11 expression based on RPKM values from RNA-sequencing studies^19^. Of the 113 patients with high FBXO11 expression, 33 featured a mutation in one of the four most common RNA spliceosome genes *SRSF2*, *SF3B1*, *U2AF1*, and *ZRSR2*, compared to 12 variants in the low FBXO11 quartile (Fig. 2d). This difference was almost entirely driven by enrichment of *SRSF2* variants in the high FBXO11 expression cohort (Fig. 2e, 21 vs. 4). The lower-than-expected number of RNA spliceosome variants in low FBXO11-expression patients suggests that FBXO11 loss contributes to MDS transformation through overlapping mechanisms.

### FBXO11 loss leads to transcriptional changes in proliferation, differentiation, and survival pathways

**Fig. 3:**
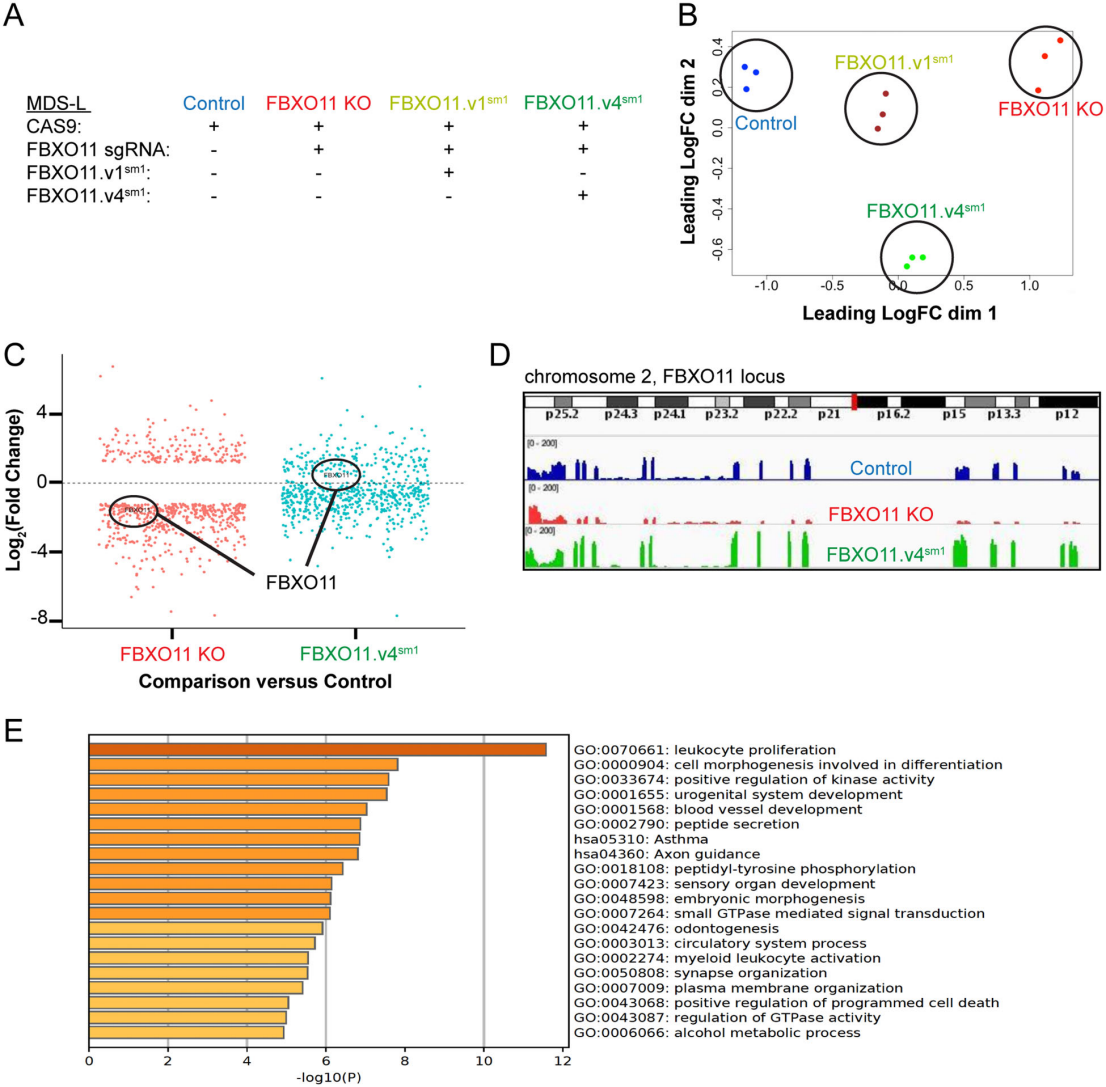
FBXO11 transcriptional networks regulate leukocyte proliferation, differentiation, and regulation of cell death. **a** Characteristics of control, FBXO11 KO, FBXO11.v1^sm1^, and FBXO11.v4^sm1^ MDS-L cells. **b** Multidimensional scaling plot showing the principal component analysis of the RNA-seq data for three independent biological replicates. **c** Scatterplot comparing fold-change values of the 677 differentially expressed transcripts in FBXO11 KO and FBXO11.v4^sm1^ conditions. Fold change is calculated compared to control MDS-L cells. FBXO11 itself is circled. **d** Tracings of the FBXO11 locus with levels of RNA detected by RNA-seq. Note the depletion of FBXO11 transcripts in FBXO11 KO and its restoration in FBXO11.v4^sm1^ cells. **e** Metascape GO pathway analysis of differentially expressed genes in the RNA-sequencing dataset. The top 20 significant pathways are shown.

Given the enrichment of FBXO11 substrates in RNA processing and metabolism, we hypothesized that FBXO11 loss would lead to broad RNA expression changes to impart cytokine-independent growth of MDS-L cells. To identify these downstream processes, we performed RNA sequencing on four MDS-L cell populations: (1) Cas9 only (Control), (2) Cas9 with FBXO11 sgRNA (FBXO11 KO), (3) Cas9 with FBXO11 sgRNA and expression of the CRISPR-resistant FBXO11.v1^sm1^ isoform (FBXO11.v1^sm1^), and (4) Cas9 with FBXO11 sgRNA and expression of the CRISPR-resistant FBXO11.v4^sm1^ isoform (Fig. 3a). We hypothesized that gene expression changes caused by FBXO11 loss that was restored by FBXO11.v1^sm1^ or FBXO11.v4^sm1^ would reveal specific transcriptional networks responsible for cytokine independence. Principal component analysis of the independent biological replicates documented strong correlation within the three experimental conditions (Fig. 3b). We detected 677 transcripts that fulfilled both criteria for differential expression upon FBXO11 loss and also a rescue of expression upon FBXO11.v4^sm1^ re-expression (Fig. 3c, Supplemental Table S4). This included the expected decrease in FBXO11 reads in FBXO11 KO cells, which was rescued by FBXO11.v4^sm1^ (Fig. 3d). We did not detect appreciable differences between the two FBXO11 isoforms, which correlates with our finding that expression of either isoform restores sensitivity to cytokine withdrawal in MDS-L (Supplemental Fig. S2A). We performed gene ontology enrichment analysis of the 677 differentially expressed genes and discovered a significant number of genes involved in leukocyte proliferation, differentiation, and regulation of cell death (Fig. 3e).

With the inverse correlation between SRSF2 variants and FBXO11 expression, one might expect there to be significant overlap in the cellular pathways impacted by *SRSF2* mutation and FBXO11 loss. To investigate this, we compared the differentially expressed genes from *SRSF2*-mutated AML cases (*SRSF2*^mt^) in the BEAT-AML database with our RNA-sequencing analysis of FBXO11 KO MDS-L cells (Supplemental Tables S4 and S5). We observed a significant enrichment of downregulated transcripts in *SRSF2*^mt^ AML and FBXO11-knockout MDS-L cells, and conclude that loss of FBXO11 expression may promote MDS transformation with a similar mechanism to *SRSF2* mutations (Supplemental Fig. S2B).

### Reduced FBXO11 expression results in alternative EZH2 splicing in sAML patients

**Fig. 4:**
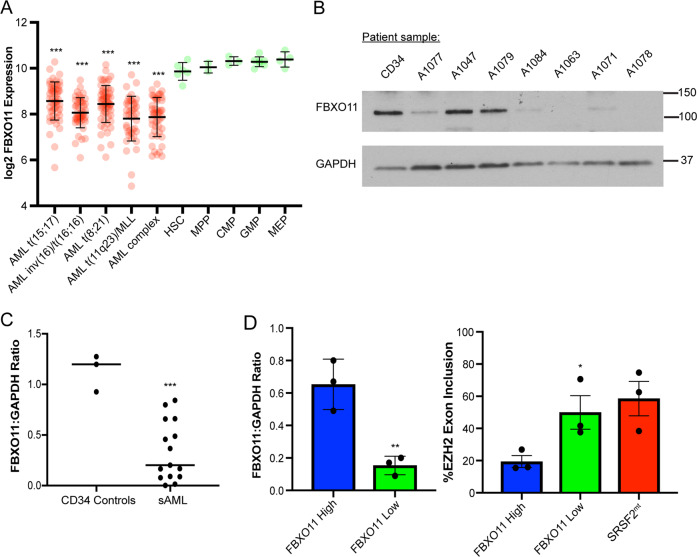
Reduced FBXO11 expression results in abnormal EZH2 splicing in patients with secondary AML. **a** Depiction of Bloodspot single-cell RNA-sequencing data for FBXO11 expression among various hematopoietic progenitor cell populations and bulk AML. An ordinary one-way ANOVA was used to test for significance between all AML subtypes and the HSC mean (***p < 0.001). **b** Representative western blot of FBXO11 levels in secondary AML samples compared to healthy CD34 cells. **c** Quantified FBXO11 expression normalized to GAPDH in 15 patients with secondary AML compared to healthy CD34 controls. An unpaired one-tailed *t* test was used to test for significance between the two groups (***p < 0.001). **d** *SRSF2*^WT^ secondary AML samples stratified by FBXO11 expression (left graph) were subsequently assayed for EZH2 exon inclusion by RT-PCR (right graph). *SRSF2*^mt^ secondary AML cases were used as a positive control for EZH2 exon inclusion. An unpaired one-tailed *t* test was used to test for significance between FBXO11-high and -low patients (*p < 0.05, **p < 0.01).

Cytokine independence is a common feature of leukemia cell lines as compared with primary cells harvested from patients with MDS. Given our observation that FBXO11-deficient MDS-L cells gained the ability to grow in the absence of cytokines, we were interested in testing whether reduced FBXO11 expression is observed in secondary AML, which would be consistent with FBXO11 downregulation being one pathway for leukemic transformation. We queried the Bloodspot single-cell RNA-sequencing database and found that FBXO11 RNA levels are decreased across many AML subtypes, including those with complex karyotype, which is commonly seen in secondary AML (Fig. 4a)^22^. To validate this finding, we prospectively collected patient leukemia samples who presented with secondary AML to the Lurie Comprehensive Cancer Center at Northwestern University. Eligible patients included those with newly diagnosed or relapsed AML with myelodysplastic changes on morphology or characteristic cytogenetics (AML–MRC), history of cytotoxic chemotherapy or radiation (tAML), or history of confirmed MDS (sAML). After purifying leukemic blasts from bone marrow aspirate or peripheral blood, we confirmed that total FBXO11 protein levels are reduced compared to healthy CD34^+^ donor cells (Fig. 4b, c). Clinical information regarding these cases is presented in Table 3.

**Table 3:** Secondary AML patient characteristics.

Patient ID	Age at diag.	Diagnosis	New or relapse	Sample info	Cytoreduction	White count	Next line of therapy	Best response	Overall survival (days)
A1052	93	AML–MRC	New	Blood	No	3.8	Aza-Ven	MFLS	287
A1053	57	sAML	Relapse	Blood	No	5.9	Aza-Ven	PD	806
A1054	71	tAML	New	Blood	No	3.8	LDC	PR	Alive
A1057	74	AML–MRC	New	Aspirate	No	1.8	Aza-Ven	MFLS	Alive
A1060	71	tAML	New	Blood	No	3.3	Dec-Ven	MFLS	108
A1068	88	AML–MRC	Relapse	Blood	Yes	17.4	Clinical trial	PD	983
A1069	61	AML–MRC	New	Blood	No	6.3	None	-	78
A1074	63	AML–MRC	New	Blood	No	3.0	LDC	CR	Alive
A1077	80	AML–MRC	New	Blood	No	5.5	Dec-Ven	PR	Alive
A1047	62	tAML	New	Blood	No	69	Dec-Ven	PD	59
A1079	83	sAML	New	Blood	No	21	Aza-Ven	PR	Alive
A1084	68	AML–MRC	New	Aspirate	No	1.2	Clinical trial	PR	Alive
A1063	70	sAML	Relapse	Blood	No	2.9	Dec-Ven	MFLS	Alive
A1071	63	tAML	New	Blood	No	9.7	LDC	CR	187
A1078	80	tAML	New	Blood	No	7.2	Aza-Ven	PD	37

Information regarding clinical samples used to generate data for Fig. 4b–d. Abbreviations: *AML—MRC* acute myeloid leukemia with myelodysplastic-related changes, *sAML* secondary AML, *tAML* therapy-related AML, *Aza-Ven* azacitidine plus venetoclax, *LDC* liposomal daunorubicin plus cytarabine, *Dec-Ven* decitabine plus venetoclax, *MFLS* morphological-free leukemia state, *PD* progressive disease, *PR* partial response, *CR* complete response. Response criteria follow ELN consensus recommendations^27^.

We were next interested in testing whether splicing alterations observed in *SRSF2*^mt^ AML cases could be detected in our patient cohort with reduced FBXO11 expression, given that *SRSF2* alterations were underpopulated in FBXO11^low^ patients in the BEAT-AML dataset. In *SRSF2*^mt^ patients, inclusion of an aberrant exon cassette containing a premature termination codon is observed in the histone methyltransferase EZH2 and results in impaired hematopoietic differentiation^20^. To assess whether this alternative splicing event occurs upon FBXO11 loss, we compared the frequency of EZH2 exon inclusion in *SRSF2*^WT^ patient secondary AML samples stratified by FBXO11 expression (Fig. 4d, left panel). We detected higher inclusion of the termination cassette in patients with low FBXO11 expression that was comparable to *SRSF2*^mt^ secondary AML controls (Fig. 4d, right panel). From these data, we conclude that downregulation of FBXO11 is observed in secondary and tAML patient samples, and patients with low FBXO11 expression have a higher incidence of abnormal EZH2 splicing events that are observed in *SRSF2*^mt^ AML.

## Discussion

In this study, we performed a genome-wide CRISPR-knockout screen for cytokine-independent survival of the MDS-L human cell line and identified FBXO11 as a potential tumor suppressor in MDS and secondary AML. We pursued FBXO11 as our lead candidate as its inactivation has been described in DLBCL and Burkitt’s lymphoma, two aggressive lymphomas of germinal-center origin^10,11^. However, the role of FBXO11 in myeloid malignancies remains unclear. This past year, emerging evidence from exome and RNA-sequencing data suggests that *FBXO11*-inactivating mutations occur in de novo AML^12^, supporting our model of FBXO11 as a tumor suppressor in myeloid malignancies. Targeted *FBXO11* sequencing of patients with MDS or secondary AML has not been performed, so it is possible that *FBXO11* mutations are more highly enriched in these patients relative to de novo AML cases. Epigenetic regulation of FBXO11 expression may also be a mechanism for downregulation in cases where no sequence alterations are present. While we did not observe a difference in the cytokine-independent phenotype of MDS-L between the larger and small isoforms of FBXO11, it is still possible that a reliance on one isoform versus the other influences the malignant potential of a hematopoietic clone.

How FBXO11 loss affects myeloid cells and contributes to leukemia progression is unclear. Interestingly, mice with germinal-center-specific knockout of *Fbxo11* develop a lymphoproliferative disease characterized by an increased number of B cells, which is likely a consequence of increased BCL6 stability^23^. It is possible that a similar myeloproliferative phenotype would be detected with FBXO11 loss in the myeloid lineage, particularly if in the background of common MDS mutations, for example in *TET2* and *DNMT3A*.

Whether the FBXO11 pathway can be therapeutically targeted will require a greater understanding of its tumor-suppressor mechanism. Our finding that spliceosome mutations are less common in patients with low FBXO11 expression raises the possibility that agents which interfere with RNA splicing might have preferential toxicity to these cells. Since FBXO11 has been demonstrated to mediate ubiquitination of BCL6 in lymphoma, it is reasonable to attempt to rescue FBXO11 loss through inhibition of proteasomal degradation. While bortezomib has shown promising response rates in combination with chemotherapy in early phase studies, randomized trials with this proteosome inhibitor have not been able to confirm clinical benefit^24,25^. Perhaps, this is due to the complete inhibition of proteosome activity rather than the ubiquitinated proteome of the FBXO11.

We surmise that FBXO11 loss will lead to the increased expression and stabilization of its candidate substrates, thus providing a rationale for small-molecule inhibitors that target these downstream pathways. However, a noncanonical F-box function should also be considered to explain its tumor-suppressor mechanism as well. As an example, FBXO11 has been shown to interact with p53 and promote its neddylation but not ubiquitination^26^. Therefore, while ubiquitination of BCL6 is a clear mechanism of tumorigenesis in lymphoid malignancies, a different pathway could explain the survival phenotype observed in the MDS-L model.

Taken together, our work implicates FBXO11 as a strong candidate tumor suppressor in MDS and sAML. Our observation that FBXO11 levels are reduced in secondary and tAML specimens provides a rationale for further in vivo modeling of FBXO11 loss in AML models and in the development of strategies to overcome its deficiency.

## Supplementary information

Supplemental figure legends and figures

Supplemental Table 1

Supplemental Table 2

Supplemental Table 3

Supplemental Table 4

Supplemental Table 5
