# Supplementary material for: FBXO11 is a candidate tumor suppressor in the leukemic transformation of myelodysplastic syndrome
Source: Blood Cancer J. 2020 Oct 6;10(10):98. doi: 10.1038/s41408-020-00362-7 (PMC7538974; doi:10.1038/s41408-020-00362-7)
Supplement: Supplementary file 1 — Supplemental figure legends and figures [file 41408_2020_362_MOESM1_ESM.pdf]

Supplemental Information

Supplemental Figures

Supplemental Figure S1: CRISPR screen in MDS-L identifies FBXO11 as a candidate tumor suppressor.

Supplemental Figure S2: RNA sequencing of FBXO11 KO MDS-L cells.

Supplemental Tables

Supplemental Table S1: Oligonucleotides used in this manuscript.

Supplemental Table S2: Raw data for CRIPSR/Cas9 Screen.

Supplemental Table S3: Raw data for 129 peptides displaying loss of ubiquitin signature in FBXO11 knockout MDS-L cells.

Supplemental Table S4: Raw data for RNA sequencing experiment.

Supplemental Table S5. Raw data for *SRSF2*^{mt} BEAT AML expression analysis.

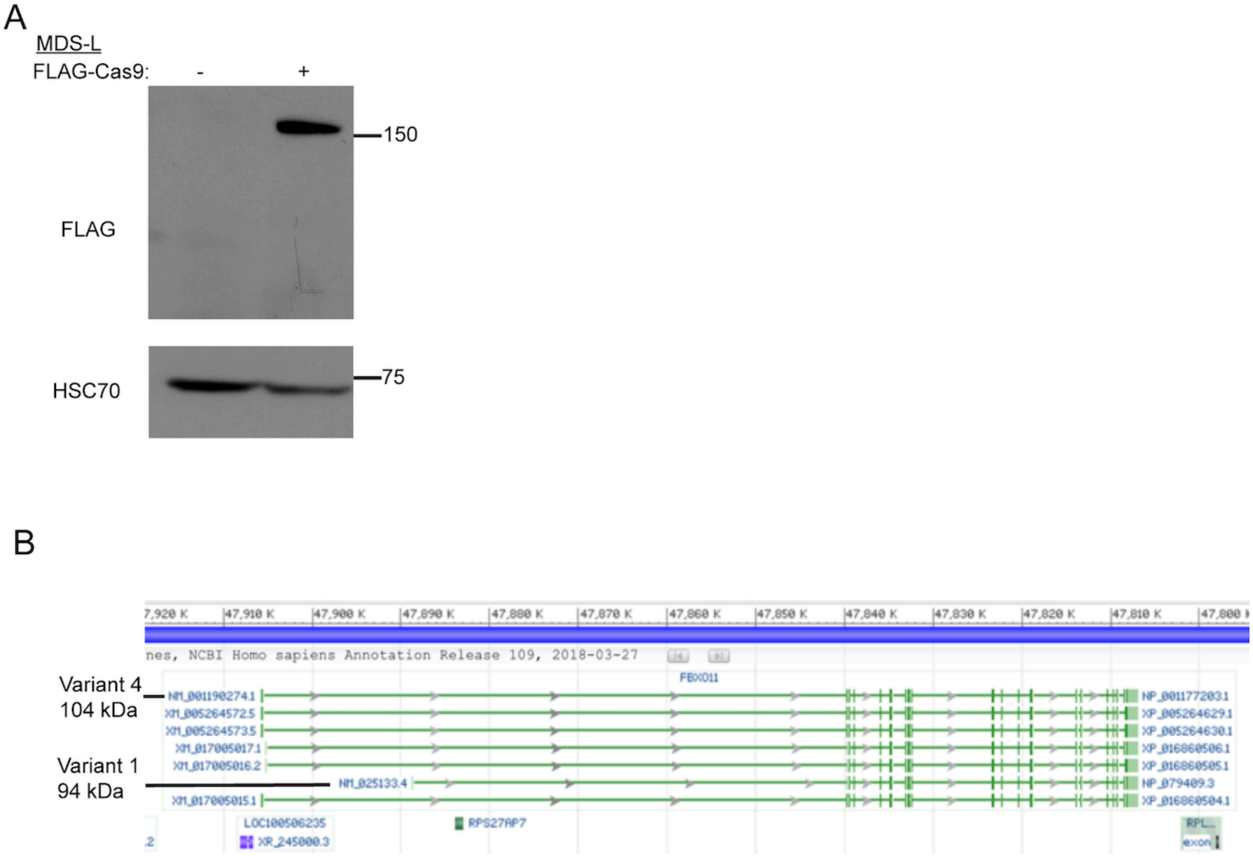

Supplemental Figure S1: **CRISPR screen in MDS-L identifies FBXO11 as a candidate tumor suppressor**. (A) Western blot confirming expression of Cas9 in MDS-L cells. (B) NCBI UniProt predicted sequences for FBXO11 include the longer isoform 4 and shorter isoform 1.

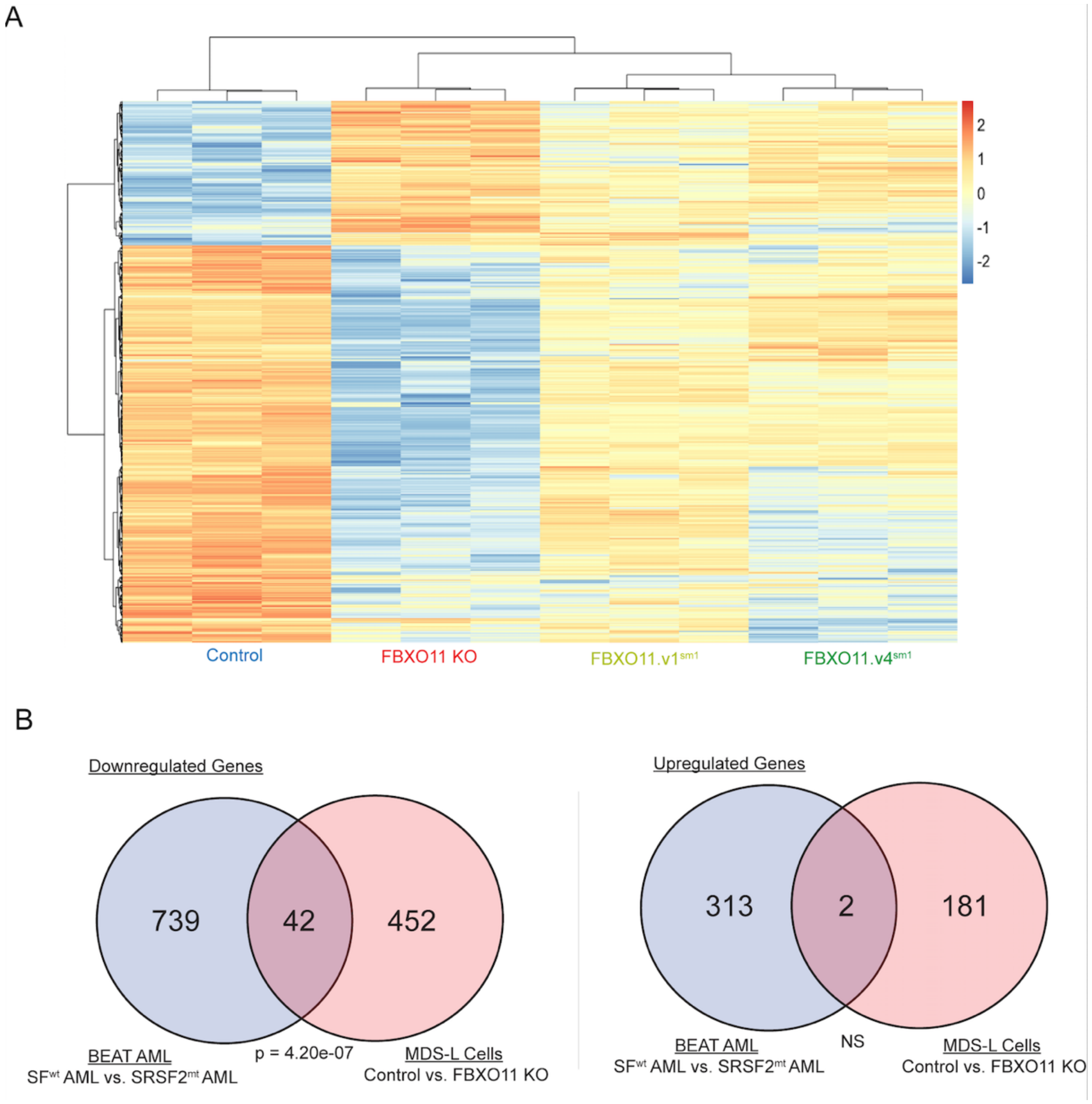

Supplemental Figure S2: **RNA sequencing of FBXO11 KO MDS-L cells**. (A) Heat map of 677 differentially expressed candidate genes from RNA sequencing. Upon FBXO11 knockout, a larger proportion of genes are downregulated (change from red to blue in FBXO11 KO) than upregulated (change to blue to red in FBXO11). The majority of these genes show normalization of their expression with reconstitution of either FBXO11 isoform. (B) Differentially expressed genes in FBXO11 knockout cells were compared to differentially expressed genes in *SRSF2*^{mt} AML cases from the BEAT AML dataset. Enrichment between the two sets was calculated using Fisher's exact test (NS = not significant).
